# Anti‐Browning and Prebiotic Effects of White Tea Extract on Fresh‐Cut Lotus Root

**DOI:** 10.1002/fsn3.72464

**Published:** 2026-10-09

**Authors:** Jingfang Li, Chuangkai Xing, Fan Jiang, Xiaoru Liu

**Affiliations:** ^1^ College of Life Sciences and Food Engineering Hebei University of Engineering Handan Hebei China; ^2^ Henan University Kaifeng Henan China; ^3^ State Key Laboratory of Food Science and Resources Nanchang University Nanchang Jiangxi China

**Keywords:** browning mechanism, lotus root, PPO inhibition, prebiotic benefits, white tea extract

## Abstract

The enzymatic browning of fresh‐cut lotus root, mediated by polyphenol oxidase (PPO), severely limits its shelf life and commercial value. This study investigated the molecular mechanism of lotus root browning and evaluated the prebiotic potential of lotus root treated with white tea (WT) extract. In the browning pattern of fresh‐cut lotus root slices, L* and b* values exhibited the most rapid changes within the first 6 h of browning, whereas the a* value increased notably after 24 h. The PPO responsible for this browning process was identified as sequence D6C6L2, and its three‐dimensional (3D) structure was established. The catechin and caffeic acid were identified as the specific substrates of PPO with lowest *K*_m_ and highest affinity, and molecular docking further revealed that these two substrates bound to the active sites of PPO via 8 and 5 hydrogen bonds, respectively. Based on these mechanistic insights, white tea (WT) extract, rich in flavanol alkaloids, was applied and found to effectively inhibit PPO activity and delay browning. In an in vitro fecal fermentation model, lotus root treated with WT extract enhanced the diversity of gut microbiota, reduced the *Firmicutes/Bacteroidetes* (*F/B*) ratio, and enriched the intestinal probiotics *Bifidobacterium*. KEGG analysis revealed that WT extract modulated carbohydrate metabolism and glycan biosynthesis pathways, suggesting potential prebiotic benefits. This study provided a scientific foundation for the development of lotus root products with both fresh‐keeping characteristics and probiotic functions.

## Graphical abstract

PPO‐driven browning limits the shelf life of fresh‐cut lotus root. Lotus root PPO was identified and its 3D structure modeled, with catechin and caffeic acid as specific substrates binding via 8 and 5 hydrogen bonds, respectively. White tea extract inhibited PPO and delayed browning of lotus root slices. White tea‐treated lotus root exhibited prebiotic potential by enhancing microbiota diversity, reducing *Firmicutes/Bacteroidetes* ratio, enriching *Bifidobacterium*, and modulating carbohydrate and glycan metabolism.

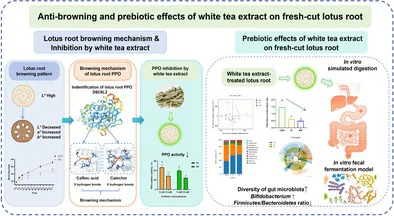

**Abbreviations**
3D: three‐dimensional
CON: control
etc‐pyrrolidinone A: (−)‐6‐(5‴*S*)‐*N*‐ethyl‐2‐pyrrolidinone‐epigallocatechin‐*O*‐gallate
etc‐pyrrolidinone B: (−)‐6‐(5‴*R*)‐*N*‐ethyl‐2‐pyrrolidinone‐epigallocatechin‐*O*‐gallate
etc‐pyrrolidinone C: (−)‐8‐(5‴‐*S*)‐*N*‐ethyl‐2‐pyrrolidinone‐epigallocatechin‐3‐*O*‐gallate
etc‐pyrrolidinone D: (−)‐8‐(5‴‐*R*)‐*N*‐ethyl‐2‐pyrrolidinone‐epigallocatechin‐3‐*O*‐gallate
*F/B*
: *Firmicutes/Bacteroidetes*
HPLC: High‐Performance Liquid Chromatography
*K*_m_
: Michaelis constant
LDA: linear discriminant analysis
LEfSe: linear discriminant analysis effect size
pI: isoelectric point
POD: peroxidase
PPO: polyphenol oxidase
ROS: reactive oxygen species
SCFAs: short chain fatty acids
SD: standard deviation
*t*_R_
: retention times
U: unit
Vc: vitamin C
WT: white tea

## 1 Introduction

Lotus root ( *Nelumbo nucifera* Gaertn.), the edible rhizome of an aquatic plant belonging to the Nymphaeaceae family. It has been cultivated and consumed for thousands of years with significant economic and cultural value in several Asian countries, particularly China, Japan, Thailand, and India (Sun et al. 2025). Lotus root is highly prized for its crisp texture and rich nutritional profile, which includes starch, dietary fiber, vitamins, minerals, and various bioactive phenolic compounds (Li, Zheng, et al. 2025; Zheng et al. 2024; Zhu 2017). In recent decades, the global demand for fresh‐cut vegetables has surged dramatically, driven by changing consumer lifestyles that prioritize convenience, freshness, and nutritional quality (Ma et al. 2017). However, post‐processing browning remains a persistent challenge for fresh‐cut lotus root. When lotus root tissues are subjected to cutting, peeling, or slicing, the exposed surfaces rapidly undergo discoloration. The enzymatic browning of lotus root severely deteriorates its visual appeal, sensory quality, nutritional value, and consumer acceptance, thereby significantly limiting its shelf life and commercial potential (Chen et al. 2026; Faisal et al. 2025). The browning problem is particularly prominent in fresh‐cut lotus root, and caused considerable economic losses and potential safety risks during its postharvest storage and processing (Guo et al. 2026).

The browning in fresh‐cut lotus root primarily attributes to enzymatic reactions. Upon tissue disruption caused by cutting or slicing, cellular compartmentalization is broken, allowing PPO to localize in plastids and cell walls to come into contact with phenolic substrates stored in vacuoles (Yuan et al. 2025). Enzymatic browning in fresh‐cut lotus root is primarily initiated by the oxidation of endogenous phenolic compounds catalyzed by PPO. Under normal physiological conditions, PPO is compartmentalized within chloroplasts and other organelles, physically separated from its phenolic substrates. However, mechanical slicing during fresh‐cut processing disrupts this cellular compartmentalization, allowing PPO to come into contact with phenolic substrates such as catechins and caffeic acid. In the presence of oxygen, PPO catalyzes the hydroxylation of monophenols to *o*‐diphenols and subsequently oxidizes *o*‐diphenols to corresponding *o*‐quinones (Zhang 2023). These highly reactive quinone compounds undergo non‐enzymatic polymerization, ultimately leading to the accumulation of dark‐colored melanin pigments on the cut surface. Given that PPO catalyzes the rate‐limiting step in this oxidative cascade, it is widely recognized as the primary enzymatic target for controlling browning in fresh‐cut fruits and vegetables. Moreover, the enzymatic browning process mediated by PPO is accompanied by a reduction in nutritional quality. Previous studies demonstrated that browning reactions significantly decrease total phenolic content and antioxidant capacity (Derardja et al. 2022). Specifically, PPO catalyzes the oxidation of phenolic compounds to reactive quinones, which subsequently undergo condensation reactions with amino acids, proteins, and other cellular constituents, leading to the loss of both nutritional and sensory attributes (Tinello and Lante 2018). Consequently, the diminished antioxidant capacity may compromise the potential health benefits of the product for consumers.

The enzymatic activity PPO correlated with the significant conformational changes (Liu, Zheng, et al. 2024). The binding process could trigger the conformational changes of PPO and substrates, which make substrates proper fitting into the active hydrophobic pocket of PPO (Li et al. 2021). The nature of PPO substrate specificity has remained elusive despite years of study (Mclarin and Leung 2020). The previous molecular docking study found that for the lotus root variety “honghuhonglian,” (+)‐catechin and (−)‐epigallocatechin are the most specific substrates of PPO, and PPO formed more hydrogen bonds and Pi‐Alkyl interactions with (−)‐epigallocatechin, while (+)‐catechin entered the active cavity of PPO more quickly due to its smaller structure (Li et al. 2023). Due to the differences of producing areas and varieties, there are differences in PPO and substrate specificity; thus, it is still necessary to further study its browning mechanism.

The inhibition strategies have been developed to combat browning and extend the shelf life of fresh‐cut lotus root, which are broadly categorized into physical, chemical, and biological methods (Sui et al. 2023). The main principles of physical techniques including low‐temperature storage, ultrasonic assisted processing, and modified atmosphere packaging, etc., work by lowering the temperature to inhibit enzyme activity, disrupting enzyme structure, or isolating oxygen, so as to prevent the browning reaction of PPO (Ali et al. 2016; Lu et al. 2026; Wen et al. 2021), while their effectiveness is often transient and insufficient for long‐term storage (Sui et al. 2023). Chemical inhibitions typically involve the use of anti‐browning agents such as citric acid, calcium chloride, and sulfites (Du et al. 2025; Shi, Xie, Sun, et al. 2024; Xiao et al. 2025). These compounds function through various mechanisms, including lowering pH, competitively inhibiting PPO, chelating copper ions at the active sites of PPO, or reducing *o*‐quinones back to colorless *o*‐diphenols. The organic acid combinate with physical processing techniques is one of the most viable approaches for PPO inhibition (Zhou et al. 2020). Sulfites are widely recognized as effective inhibitors of PPO, nevertheless, their applications are limited by documented adverse reactions, including asthma exacerbations, anaphylaxis, and urticaria in susceptible individuals (Li et al. 2024). Biological methods have attracted increasing attention, including the application of plant derived natural inhibitors, microbial metabolites and other active compounds. *Saccharomyces cerevisiae* extracts significantly reduced the browning of fresh‐cut vegetable and fresh juice (Liu, Ding, et al. 2024; Liu, Li, et al. 2024; Liu, Zheng, et al. 2024). The core inhibitory mechanism was that glutathione in the extract interacted with non‐active sites of PPO, leading to the formation of stable GSH‐PPO‐substrate complexes and affecting PPO activity. The seabuckthorn leaf extract significantly inhibited the browning of fresh‐cut potatoes by its gallic acid stably bound to the active site of PPO (Zhang et al. 2022). Gallic acid in seabuckthorn leaf extract bound to the active sites of PPO, significantly inhibiting browning in fresh‐cut potatoes. Green tea extract was an effective anti‐browning agent for short‐time stored cloudy apple juices (Klimczak and Gliszczyńska‐świgło 2017). The key bioactive components responsible for this inhibitory effect and their specific mechanisms remain to be fully elucidated. In recent years, natural plant extracts rich in polyphenols and their derivatives have attracted increasing attention as safer and more sustainable alternatives. However, research on the anti‐browning potential of natural extracts, particularly WT extract, on lotus root remains limited. Considering the limitations of existing commonly used PPO browning inhibitors, there is a clear need to identify effective, low‐cost, and natural anti‐browning compounds that meet consumer demand for clean‐label products. Tea is rich in flavanol derivatives, particularly in WT, which contain components such as flavanol alkaloids (Li et al. 2018). Previous studies demonstrated that flavanol derivatives exhibited various biological activities, including antioxidant, anti‐aging, acetylcholinesterase inhibition effect, and suppression of advanced glycation end product formation (Gaur et al. 2020; Ke et al. 2024; Li et al. 2018; Wang et al. 2014). The prior research found that PPO in lotus root has a strong affinity for flavanol compounds, such as catechin and epigallocatechin. Therefore, investigating flavanol derivatives with similar structures as potential inhibitors and exploring their inhibitory effects on browning in fresh‐cut lotus root holds significant research value.

Extensive research has focused on understanding and controlling browning in lotus root, while the preservation technologies are typically optimized solely for visual quality maintenance and shelf‐life extension of lotus root, without consideration of their impact on the functional properties and potential health benefits. Previous study confirmed lotus root is rich in diverse bioactive components that confer upon it the potential to modulate the gut microbiota (Guan et al. 2022). Nevertheless, various processing and browning inhibitor treatments applied to lotus root may cause unpredictable changes in their efficacy in regulating the gut microbiota (Zhao et al. 2024). Concurrently, WT has attracted increasing attention for its capacity to modulate gut microbiota. WT was reported to exert prebiotic‐like effects by promoting the growth and colonization of beneficial bacterial families, including *Bifidobacterium* and *Akkermansia*, while suppressing potentially harmful taxa (Zhou et al. 2022; Lin et al. 2024). In particular, the impact of browning inhibitor treatments on the bioactive constituents of lotus root, and consequently on their capacity to modulate gut microbiota, remains largely underexplored. As various processing steps and inhibitor applications may alter the structural integrity, chemical composition, and intestinal bioavailability of key bioactive compounds, potentially leading to unpredictable changes in their efficacy as prebiotic modulators. Therefore, it is essential to evaluate not only the anti‐browning efficacy of WT extract but also its impact on the prebiotic properties of lotus root.

Thus, the objectives of the present study were to (i) explore the browning patterns and mechanisms of fresh‐cut lotus root under different conditions; (ii) evaluate the inhibitory effect of WT extract on browning in fresh‐cut lotus root slices and how these inhibition treatments influence the capacity of lotus root to modulate the gut microbiota. The findings are expected to strengthen lotus root browning control that delivers products which are not only visually appealing but also retain their gut health‐promoting potential, as well as offer valuable insights for the fresh‐cut produce industry.

## 2 Materials and Methods

### 2.1 Plant Materials and Chemical Reagents

Fresh Lotus roots (*Nelumbo.nueifera* cv. “Cizhou Bailian”) were obtained from Handan (Hebei, China). Fresh Lotus roots were cut into 3–5 mm thin slices and were placed in the sterilized constant temperature and humidity incubator (HPX‐160BSH, Xinmiao, China) for further test.

All standards including caffeic acid, 5‐*p*‐coumaroylquinic acid, shikimic acid, rutin, proanthocyanidin B3, epigallocatechin, catechin, epicatechin, dimethyl ellagic acid, swertianolin, esculin were obtained from Sigma‐Aldrich (St. Louis, Mo, USA). Folin‐Ciocalteau reagent, High‐Performance Liquid Chromatography (HPLC)‐grade acetonitrile, methanol, and formic acid were obtained from Merck (Darm Stadt, Germany). The Vitamin C (Vc) and other chemical reagents were analytical grade purchased from Xilong Scientific (Guangdong, China). The white tea Bai‐Mudan was purchased from Fujian Pinpinxiang Tea Co. Ltd. (Fujian, China). Water was purified using a Milli‐Q system from Millipore (Bedford, Ma, USA).

### 2.2 Browning Index

The surface color of sliced lotus root was assessed employing a chromameter (CR‐10, Konica Minolta, Japan). Color coordinates were recorded in the CIE L*, a*, and b* color space, representing lightness, red‐green, and yellow‐blue components, respectively. Additionally, the browning index, an indicator of browning extent, was calculated during storage based on the following equation (Ergunes and Tarhan 2006; Nourzad et al. 2024):

$$\mathrm{BI}=\left[100\left(x-0.31\right)\right]/0.172,\text{where}\;x=\left(a+1.75L\right)/\left(5.645L+a-3.012b\right).$$

### 2.3 Extraction, Purification, and Identification of PPO From Lotus Root

PPO from lotus root was extracted, purified, and identified following a previously reported method with minor modifications (Li et al. 2023). In brief, 100 g of lotus root was homogenized (Thermo Fisher Scientific, Waltham, MA, USA) in 3000 mL of PBS (20 mM, 1% PVPP, 0.15% Triton X‐100) for 60 s and then continuously stirred at 4°C for 6 h. The homogenate was centrifuged at 5000 rpm for 15 min at 4°C and the supernatant was precipitated with 80% saturated ammonium sulfate, followed by centrifugation at 5000 rpm for 20 min at 4°C. The sediment was collected and dialyzed until free of ammonium sulfate to obtain the crude PPO.

The purification of PPO from lotus root was performed following the method of Han et al. (2019) with minor modifications. In detail, the crude PPO was first purified by anion‐exchange chromatography on a DEAE‐Sepharose Fast Flow column (GE Healthcare Bio‐Science AB, Uppsala, Sweden), which was pre‐equilibrated with 20 mM Tris–HCl buffer (pH 7.2). The protein was eluted with a linear NaCl gradient (0–0.5 M) at a flow rate of 1 mL/min. Fractions were collected and monitored for UV absorbance (Shanghai Precision Scientific Instrument Co. Ltd., Shanghai, China) at 280 nm. Those exhibiting PPO activity were pooled, concentrated by 10 kDa ultrafiltration, and applied to a Sephadex G‐75 gel filtration column (1.6 × 100 cm, GE Healthcare Bio‐Science AB, Uppsala, Sweden) for further purification. The column was equilibrated and eluted with 20 mM of Tris–HCl buffer (pH 7.2) at a flow rate of 0.3 mL/min. The fractions with high PPO activity were collected and concentrated for PPO identification.

The purified PPO was loaded onto SDS‐PAGE in a Mini Protean III Electrophoresis Cell (Bio‐Rad Laboratories, Hercules, CA, USA) and target protein bands were excised, subjected to in‐gel tryptic digestion, and the extracted peptides were analyzed by Q Exactive LC–MS/MS (Thermo Fisher Scientific, Waltham, MA, USA). Chromatographic separation was performed on a nano‐Viper C18 column (100 μm × 100 mm, 100 Å, Thermo Fisher Scientific, Waltham, MA, USA) with an acetonitrile gradient. Proteins were identified by searching the MS/MS data against the *N. nucifera* database (UniProt database, https://www.uniprot.org/).

### 2.4 Extraction and Identification of Phenolic Compounds From Lotus Root

The phenolic compounds from lotus root were extracted and identified according to the protocol described by Wu et al. (2021) and Zhu et al. (2022) with slight modifications.

#### 2.4.1 Ultrasound Assisted Extraction

Lotus root samples were freeze‐dried (FD‐1, Beijing Detianyou Technology and Development Co. Ltd., Beijing, China) to constant weight and ground into a fine powder that passed through a 100‐mesh sieve. The powder of lotus root (30.00 g) was extracted with 600 mL of 80% methanol (v/v) with a solid‐to‐liquid ratio of 1:20 (w/v). Ultrasonic‐assisted extraction (Greenapple, Shanghai, China) was carried out at 300 W and 20 kHz for 30 min at 40°C. After extraction, the mixture was centrifuged at 4200 rpm for 10 min at room temperature (Thermo Scientific, Waltham, MA, USA), and the supernatant was collected. The residue was re‐extracted twice more with fresh 80% (v/v) methanol under the same conditions. All supernatants were concentrated using a rotary evaporator (Eyela N‐100, Japan) until a paste was obtained. The evaporation was conducted at 45°C with a rotation speed of 80 rpm under the pressure of 0.09 MPa. The concentrate was redissolved in 10 mL of anhydrous methanol and filtered through a 0.22 μm nylon membrane.

#### 2.4.2 Identification of Phenolic Compounds by HPLC‐ESI‐QTOF‐MS/M

The phenolic compounds in the lotus root extract were analyzed using an Agilent 1260 HPLC system (Agilent Technologies, Santa Clara, USA) equipped with a DAD detector and coupled to an Agilent 6538 Accurate‐Mass Q‐TOF mass spectrometer (Agilent Technologies, Santa Clara, USA). An Agilent Zorbax Eclipse Plus C18 column (4.6 × 250 mm, 5 μm) was used at 30°C. The mobile phase consisted of 0.1% (v/v) formic acid in water (A) and methanol (B) at a flow rate of 0.3 mL/min. The gradient elution program was as follows: 0–15 min, 5%–25% B; 16–35 min, 25%–55% B; 36–55 min, 55%–80% B; 55–60 min, 80%–100% B. The injection volume was 3 μL, and the UV chromatogram (Shanghai Precision Scientific Instrument Co. Ltd., Shanghai, China) was monitored at 280 nm.

Mass spectrometry detection was conducted in negative electrospray ionization (ESI) mode. The source parameters were set as follows: capillary voltage, 4.0 kV; fragmentor voltage, 135 V; source temperature, 100°C; collision gas, nitrogen; drying gas temperature, 350°C; drying gas flow, 10.0 L/min; nebulizer pressure, 40 psi; and collision energy, 30 eV; full scan mass range, *m/z* 50–900. Data were collected using Agilent MassHunter Workstation software (version B.03.01).

### 2.5 Substrate Specificity Assay

The kinetic parameters including Michaelis constant (*K*_m_) of lotus root PPO were determined using the *Lineweaver–Burk* method (Lineweaver and Burk 1934). The enzyme activity was assayed at a range of substrate concentrations. The calculation of *K*_m_ was shown as the following formula (Han et al. 2019).

$$v=V_{\max}\left[S\right]/\left(K_{m}+\left[S\right]\right)$$

$$1/v=K_{m}/\left[S\right]V_{\max}+1/V_{\max}$$

where *v* is the reaction rate and [*S*] is the substrate concentration.

### 2.6 3D Structure of PPO and Molecular Docking

The 3D structure of lotus root PPO and molecular docking analysis were performed following the method described by Liu, Ding, et al. (2024). The amino acid sequences of the purified PPO, which showed high similarity to *N. nucifera* PPOs in the UniProt database, were used to generate the 3D structure of lotus root PPO via homology modeling using the Swiss‐Model server (Swiss‐Model, https://www.swissmodel.expasy.org). The resulting model with the highest Profiles‐3D score was selected for subsequent analysis. The chemical structures of the substrates were acquired from the Zinc database (https://zinc.docking.org).

Molecular docking was performed using AutoDock 4.2 to investigate the interactions between PPO and the substrates, including the identification of potential binding sites, interaction forces, and binding energies. For molecular docking, the grid box was centered at the catalytic active site of PPO (coordinates: *x* = 16.483, *y* = 43.179, *z* = 78.023) with dimensions of 100 × 100 × 100 Å^3^ and a grid spacing of 0.375 Å. For the molecular docking simulation of *Malus domestica* chloroplastic polyphenol oxidase MdPPO1 (PDB: 6ELS), the docking grid was constructed exclusively around the di‐copper catalytic pocket. According to the study of Kampatsikas et al. (2019), the C‐terminal segment spanning residues 371–593 functions as an autoinhibitory shielding domain that sterically blocks the active site in latent full‐length protein. This autoinhibitory region was entirely excluded from the docking box, since only proteolytic cleavage of residues 371–593 generates the physiologically active enzyme with an exposed ligand‐binding pocket. This setup eliminates steric interference from the autoinhibitory domain and avoids biologically irrelevant binding poses. For each ligand, at least 100 independent docking runs were conducted to adequately sample the possible binding conformations. The optimal docking pose with the lowest calculated binding energy, which implies the highest binding affinity, was selected for further analysis.

### 2.7 Assay of PPO Activity and Inhibition Processing of Fresh‐Cut Lotus Root Slices

#### 2.7.1 Assay of PPO Activity

PPO activity was assayed following the method described by Liu et al. (2025) with slight modifications. In brief, the PPO activity assay was initiated by mixing 0.4 mL of PPO enzyme solution with 5.6 mL of 0.2 M catechol (dissolved in 0.05 M PBS, pH 6.5). The increase in absorbance at 420 nm was monitored kinetically at 35°C for 3 min using a Hitachi 722G spectrophotometer (Hitachi Instruments Inc., Shanghai, China). One unit (U) of PPO activity was defined as the amount of enzyme causing an increase of 0.001 in absorbance per minute per mL of enzyme solution under the assay conditions. The activity was calculated according to the following formula:

$$\mathrm{PPO}\;\text{activity}\;\left(U\right)=\Delta A_{420}/\left(0.001\times V\times t\right)$$

where Δ*A*_420_ is the change in absorbance at 420 nm, *V* is the volume of the enzyme solution used in the assay (mL), and t is the reaction time (min).

The soluble protein content in the enzyme extracts was determined using the Bradford method (Bradford 1976). The specific activity of PPO was subsequently calculated by dividing the total PPO activity by the total protein concentration (mg/mL) of the corresponding extract, and was expressed as units per milligram of protein (U/mg).

#### 2.7.2 Inhibition Processing of Fresh‐Cut Lotus Root Slices

The method of extracting flavanol derivatives from WT referred to the study of Li et al. (2018). In brief, WT Bai‐Mudan (1 kg) was ground and extracted 3 times with 80% acetone/water (w/w) at room temperature, and the combined extract was concentrated under reduced pressure to yield a water‐soluble extract. The extract was successively partitioned with dichloromethane, ethyl acetate, and n‐butanol (1:1, v/v each) to obtain dichloromethane‐soluble (75 g), ethyl acetate‐soluble (170 g), n‐butanol‐soluble (50 g), and water‐soluble residual fractions (158 g). The n‐butanol fraction was subjected to repeated column chromatography over Sephadex LH‐20 (GE Healthcare Bio‐Sciences AB, Stockholm, Sweden), and 4 flavoalkaloids were mainly obtained, including (−)‐6‐(5‴*S*)‐*N*‐ethyl‐2‐pyrrolidinone‐epigallocatechin‐*O*‐gallate (ester‐type catechins pyrrolidinone A, etc‐pyrrolidinone A), (−)‐6‐(5‴*R*) ‐*N*‐ethyl‐2‐pyrrolidinone‐epigallocatechin‐*O*‐gallate (etc‐pyrrolidinone B), (−)‐8‐(5‴‐*S*)‐*N*‐ethyl‐2‐pyrrolidinone‐epigallocatechin‐3‐*O*‐gallate (etc‐pyrrolidinone C) and (−)‐8‐(5‴‐*R*) ‐*N*‐ethyl‐2‐pyrrolidinone‐epigallocatechin‐3‐*O*‐gallate (etc‐pyrrolidinone D). The efficacy of inhibitors on fresh‐cut lotus root slices and the activity of PPO was conducted following the method of Han et al. (2019) with minor modifications. The effects of inhibitors including WT extract and Vc on the activity of lotus root PPO were evaluated at concentrations of 5 mM and 10 mM. The residual PPO activity was measured at 35°C, and the results are expressed as the percentage of initial PPO activity without inhibitors. The PPO relative activity was calculated using the following formula:

$$\mathrm{PPO}\;\text{relative activity}\;\left(\%\right)=\left(1–U_{2}/U_{1}\right)\times 100$$

where *U*_2_ represents the enzyme activity of the inhibition group (U/g), and *U*_1_ represents initial PPO activity without inhibitors (U/g).

### 2.8 In Vitro Simulated Digestion and Colonic Fermentation

#### 2.8.1 Oral Digestion

The in vitro simulated digestion protocol was adapted from Brodkorb et al. (2019) with minor modifications. Lotus root slices were treated with WT (10 mM) and Vc (10 mM) respectively, followed by freeze‐drying and sieving into a fine powder (100‐mesh sieve). Briefly, crushed lotus root samples (20 g, pretreated with inhibitors) were first subjected to oral digestion by mixing with simulated saliva fluid (17 mL), salivary α‐amylase (1 mL, 1000 U/mL), CaCl_2_ (50 μL, 0.3 M), and distilled water (1.95 mL). The mixture was incubated in a shaking water bath (Thermo Fisher Scientific, Waltham, MA, USA) at 37°C for 2 min.

#### 2.8.2 Gastric Digestion

After oral digestion, 20 mL of the oral digesta was transferred to a gastric digestion system. The simulated gastric fluid (15 mL), porcine pepsin stock (2 mL, 1500 U/mL), gastric lipase (1.2 mL, 60 U/mL). The pH was then lowered to 3.0 by incorporating 1 M HCl, and the volume was then brought up to 40 mL with ultrapure water. The resulting mixture was incubated for 2 h at 37°C under constant agitation.

#### 2.8.3 Intestinal Digestion

After completing the gastric phase, 40 mL of the gastric digest was used for intestinal digestion. This aliquot was combined with 22 mL of simulated intestinal fluid, 10 mL of porcine pancreatin (800 U/mL), 5 mL of cholic acid, 80 μL of 0.3 M CaCl_2_, and 2.62 mL of water. The pH of the blend was subsequently adjusted to 7.0 with 1 M NaOH, and the volume was then brought up to 70 mL with ultrapure water, followed by a 2 h incubation at 37°C in a shaking bath (Thermo Fisher Scientific, Waltham, MA, USA).

#### 2.8.4 In Vitro Colonic Fermentation

The in vitro colonic fermentation procedures were carried out based on the protocol of Xia et al. (2025) with slight modifications. Following in vitro simulated digestion, the processed lotus root samples were incubated under anaerobic conditions for colonic fermentation. The fermentation was performed in an anaerobic chamber maintained at 37°C with an atmosphere composed of 80% N_2_, 10% H_2_, and 10% CO_2_. Human feces served as the microbial inoculum, obtained from 6 healthy adult volunteers aged 25–40 years, including 3 males and 3 females with a body mass index below 24.0. These donors reported no use of antibiotics or antifungal agents within 3 months prior to feces sample collection. The freshly voided feces were immediately mixed with anaerobic medium under anaerobic conditions to obtain a 5% (w/v) fecal suspension, which was subsequently passed through sterile gauze to obtain the fermentation solution. The colonic fermentation was performed for 48 h.

### 2.9 Gut Microbe 16S rRNA Sequencing

The gut microbial 16S rRNA sequencing was performed following methods of Liu et al. (2025) with modifications. Following colonic fermentation of lotus root samples, microbial genomic DNA was isolated with the Magnetic Soil and Stool DNA Kit (Tiangen Biotech, Beijing, China), and all extracted DNA samples were kept at −80°C for subsequent analysis. DNA quantification was accomplished on a Qubit 3.0 Fluorometer with the Qubit dsDNA HS Assay Kit (Invitrogen, Thermo Fisher Scientific, Oregon, USA). Amplification of the 16S rRNA gene V3–V4 region was conducted on a Hiseq platform (IL, USA) employing primers 27F (AGRGTTTGATYNTGGCTCAG) and 1492R (TASGGHTACCTTGTTASGACTT). The total of PCR amplicons was purified and quantified using Agencourt AMPure XP Beads (Beckman Coulter, Indianapolis, IN) and Qubit fluorescence depending on the preliminary electrophoresis quantitative results. Equimolar pooled and barcoded amplicons were then processed into SMRTbell libraries and loaded onto a PacBio Sequel II 8 M flow cell for sequencing with the Sequel II Sequencing Kit 2.0. Sequence data were analyzed using the BMK Cloud platform (Biomarker Technologies Co. Ltd., China). The OTU clustering (≥ 97% identity) was conducted with QIIME1, and subsequent taxonomic assignment was performed at the genus level based on OTU homology.

### 2.10 Data Analysis

Data were expressed as mean ± standard deviation (SD). All experiments were performed in triplicates. The statistical analyses were processed and plotted out using the SPSS statistical software (Version 25.0, SPSS Inc., USA). One‐way ANOVA followed by *Duncan*'s multiple range tests was performed to determine the significance of difference, and differences were considered to be significant at the *p* < 0.05 level.

## 3 Results

### 3.1 Browning Pattern of Lotus Root

The browning patterns of lotus root differed markedly under storage conditions of 4°C and 25°C, revealing that browning was not a stochastic process but followed distinct kinetics driven by a cascade of biochemical processes. As shown in Figure 1A, the browning index of lotus root increased significantly at 25°C, especially during the initial 6 h, when the rise was most pronounced. The browning degree of lotus root slices at 4°C was significantly lower than that at 25°C (*p* < 0.01). This temperature‐dependent behavior reflects the underlying enzymatic nature of the browning reaction, as PPO activity is strongly influenced by temperature. Specifically, the L* value of lotus root slices decreased significantly under both 4°C and 25°C (Figure 1B), and the most pronounced decline of lightness occurred within the first 6 h. A significant divergence in L* values between the 4°C group and 25°C group emerged after 6 h, and the difference had become highly significant by 24 h (*p* < 0.01). Consistent with the trend observed in the L* value, the b* value (Figure 1D) of lotus root slices increased significantly at both 4°C and 25°C. The most rapid increase occurred within the first 6 h at 25°C, indicated that a pronounced rise in yellowness occurred during this period, followed by a reduced rate of change. After 48 h, a highly significant difference in b* value was observed between the 4°C and 25°C conditions (*p* < 0.01). Different from the L* and b* values, the a* value (redness) of lotus root slices began to increase markedly after 24 h (Figure 1C). A highly significant difference in a* values was observed between the 4°C and 25°C groups after 48 h. The findings demonstrated that storage at 4°C significantly delayed the decrease in L* value and the increase in a* and b* values compared to 25°C. Although changes in L* and b* values were most pronounced within the first 6 h, the notable rise in a* value occurred predominantly after 24 h. PPO is recognized as the rate‐limiting enzyme in the enzymatic browning pathway of fruits and vegetables, playing a central role in initiating the oxidative cascade that leads to melanin formation. The catalytic activity of PPO is highly temperature‐dependent, making temperature management a critical factor in controlling browning progression. In addition, the occurrence of the browning reaction is not only related to PPO, but also closely related to peroxidase (POD) and reactive oxygen species (ROS), which serve as cooccurring mechanisms that synergistically accelerate browning. POD can oxidize phenolic substrates in the presence of H_2_O_2_, while ROS, generated as byproducts of wounding stress and mitochondrial respiration, can both directly oxidize phenolics and enhance PPO activity (Zhang et al. 2026). Consistent with these findings, Min et al. (2017) confirmed that the activities of PPO, POD, and their corresponding gene expressions increased in parallel with the increase in browning degree during storage at different temperatures.

**FIGURE 1:**
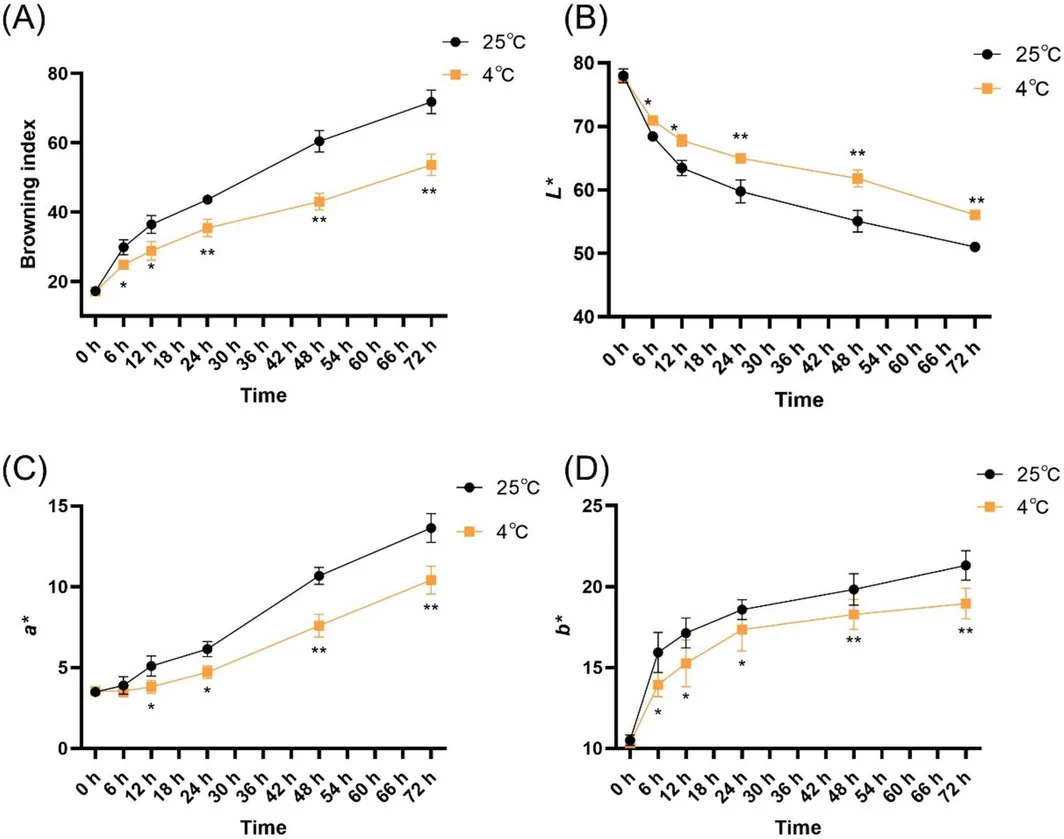
The browning patterns of lotus root at 4°C and 25°C. (A) Browning index of lotus root; (B) L* represents light–dark; (C) a* represents red‐green; (D) b* represents yellow‐blue. *p < 0.05, **p < 0.01 by the student's *t* test indicates significant differences between lotus root at 4°C and 25°C.

### 3.2 The Identification and 3D Structure Modeling of PPO From Lotus Root

The results of mass spectrometry identification for the PPO from Cizhou lotus root are presented in Table S1. The purified PPO band matched the PPO sequence (accession no. D6C6L2) in the UniProt database, supported by two unique peptides with high confidence. This PPO sequence consists of 597 amino acids, has a molecular weight of 67.22 kDa, and an isoelectric point (pI) of 8.59. The sequence D6C6L2 of purified lotus root PPO was submitted to SWISS‐MODEL for homology modeling, and the results are shown in Figure 3A. The lotus root PPO exhibited high sequence similarity with the amino acid sequence of PDB code 6ELS, with a sequence homology of 64.65%. This structure had an active center composed of one O atom and two Cu atoms. The reliability of this PPO structure was verified via Ramachandran plots (Figure S1), which indicated that 95.49% of the amino acid residues are distributed in the favored region, with only 0.43% of the amino acid residues being outliers, thus proving the reliability of the 3D structure modeling of PPO from lotus root. In a similar manner to the lily bulb PPO study, Xu et al. (2025) constructed a 3D structural model of PPO using homology modeling based on the apple‐derived PPO.

### 3.3 Analysis of Phenolic Compounds in Lotus Root

The phenolic compounds identified in lotus root are presented in Figure 2 and Table 1. A total of 16 compounds were identified based on comparisons of their retention times (*t*_R_), authentic standards, and MS/MS data from the literature. The 16 peaks were identified as caffeic acid (peak 1), caffeyl hexose (peak 2), 5‐*p*‐coumaroylquinic acid (peak 3), shikimic acid (peak 4), rutin (peak 5), hydroxyproanthocyanidin dimers B1‐OH (peak 6), hydroxyproanthocyanidin dimers B2‐OH (peak 7), protocatechuic acid hexoside (peak 9), proanthocyanidin B3 (peak 10), hydroxyproanthocyanidin dimers B4‐OH (peak 11), (−)‐epigallocatechin (peak 12), catechin (peak 13), epicatechin (peak 14), dimethyl ellagic acid (peak 15), swertianolin (peak 16), and esculin (peak 17), respectively. The substances in peaks 8 and 18 were not identified.

**FIGURE 2:**
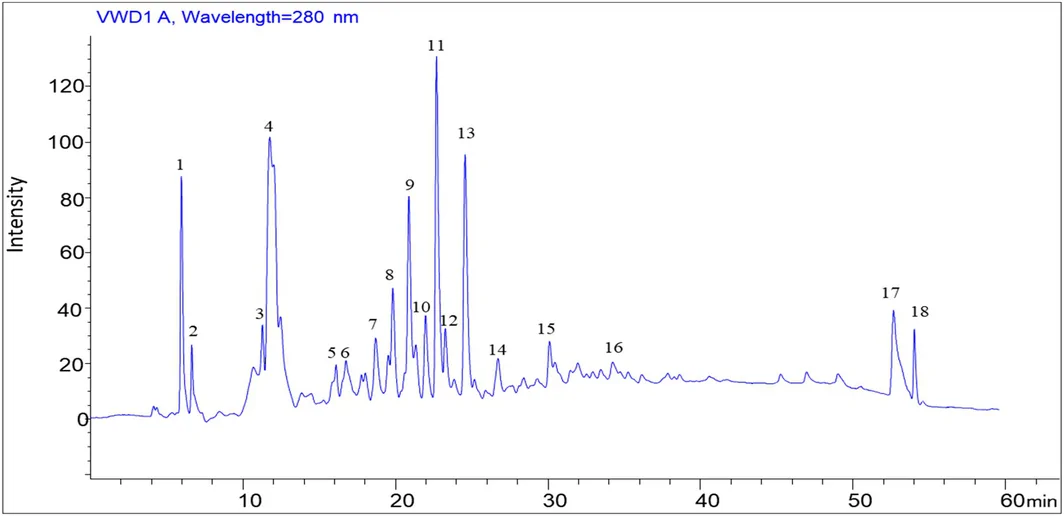
HPLC chromatogram of main phenolic compounds in lotus root.

**TABLE 1:** Qualitative analysis of main phenolic compounds in lotus root.

Peak no.	*t*_R_ (min)	[M − H]^−^ (*m/z*)		Molecular formula	Error (ppm)	Major fragment ions (*m/z*)	Tentative identification/proposed compound
		Expected	Found at				
1	6.29	179.0564	179.0566	C_9_H_8_O_4_	1.2	59.0138, 75.0089, 161.0459, 179.0573	Caffeic acid
2	7.46	341.1104	341.1103	C_12_H_22_O_11_	−0.4	161.0488, 179.0600	Caffeyl hexose
3	10.31	337.0786	337.0788	C_12_H_18_O_11_	0.6	174.0177, 277.0561	5‐*p*‐Coumaroylquinic acid
4	10.77	173.0099	173.0102	C_6_H_6_O_6_	1.5	71.0162, 96.9943, 124.9901	Shikimic acid
5	15.54	609.1274	609.1279	C_23_H_30_O_19_	0.9	—	Rutin
6	16.28	593.1322	593.1324	C_30_H_26_O_13_	0.5	—	Hyroxyproanthocyanidin dimers B1‐OH
7	17.5	593.1325	593.1327	C_30_H_26_O_13_	0.3	—	Hyroxyproanthocyanidin dimers B2‐OH
9	19.36	315.1089	315.1092	C_14_H_20_O_8_	0.8	123.0460, 153.0570	Protocatechuic acid hexoside
10	20.13	577.1379	577.1381	C_30_H_26_O_12_	0.3	407.0783, 425.0900, 289.0722, 125.0251, 161.0239, 245.0836, 287.0562, 451.1041	Proanthocyanidin B3
11	21.3	593.1316	593.1321	C_30_H_26_O_13_	0.9	—	Hyroxyproanthocyanidin dimers B4‐OH
12	22.34	305.0679	305.0677	C_15_H_14_O_7_	−0.4	125.0274, 139.0422, 137.0272, 164.0124, 165.0226, 179.0398, 221.0468, 262.0789	Epigallocatechin
13	23.25	289.0729	289.0730	C_15_H_14_O_6_	0.6	123.0468, 151.0419, 179.0379, 205.0534, 245.0858, 271.0635	Catechin
14	26.28	289.072	289.0724	C_15_H_14_O_6_	1.4	123.0443, 151.0395, 79.0337, 205.0522, 245.0814, 271.0635	Epicatechin
15	28.67	329.0885	329.0887	C_14_H_18_O_9_	0.4	123.0471, 167.0381, 191.0391, 209.0505	Dimethyl ellagic acid
16	34.62	435.1299	435.1305	C_21_H_24_O_10_	1.4	123.0451, 167.0364, 273.0775	Swertianolin
17	53.54	339.2338	339.234	C_23_H_32_O_2_	0.5	163.1132	Esculin
8	20.00	305.0681	305.0683	—	0.7	—	Unidentified
18	55.93	227.2026	227.2023	—	−1.4	—	Unidentified

The composition and content of endogenous phenolic compounds in lotus root are critical determinants of its susceptibility to enzymatic browning, while the phenolic profile varies depending on the cultivation region and variety. Previous studies have characterized the phenolic profile of lotus root, identifying chlorogenic acid, catechin, epicatechin, gallic acid, *p*‐coumaric acid 4‐*O*‐glucoside, etc. as the major phenolic components (Zhu et al. 2022). Regarding substrate specificity toward PPO, recent studies revealed that (−)‐epigallocatechin and (+)‐catechin are the key substrates of lotus root PPO (Li et al. 2023). In addition, temperature has been shown to modulate this process by upregulating PAL activity, which drives de novo phenolic synthesis and provides substrates for PPO oxidation. Collectively, these findings established that the inherent phenolic profile of lotus root was a fundamental determinant of browning potential, and that temperature can further influence browning progression through the regulation of phenolic metabolism.

### 3.4 Binding Affinities of PPO With Key Phenolic Compounds in Lotus Root

The binding energies between lotus root PPO and the major phenolic compounds were analyzed (Table S2). The results revealed that PPO exhibited the strongest binding affinity with catechin and caffeic acid, as indicated by the lowest docking energy (catechin, −6.99 kcal/mol; caffeic acid, −5.73 kcal/mol), followed by epicatechin, epigallocatechin, esculin, and dimethyl ellagic acid. The results suggested that the PPO‐catalyzed reaction with these substrates was spontaneous and the most likely to occur. Consequently, these compounds were excluded from further analysis. For other compounds identified in lotus root, the docking energies were all greater than 0, indicating that the catalytic reactions would not be spontaneous.

The major phenolic substrates in lotus root were further quantified (Table 2). The results showed that catechin was the most abundant (112.89 ± 7.94 μg/g), followed by caffeic acid (81.92 ± 5.34 μg/g). The contents of other substrates were relatively low, with epigallocatechin at 28.63 ± 2.11 μg/g, esculin at 21.38 ± 2.13 μg/g, dimethyl ellagic acid at 16.53 ± 2.78 μg/g, and epicatechin at 14.71 ± 1.36 μg/g.

**TABLE 2:** Content of main phenolic substrates and their affinity with PPO.

Main phenolic substrates	Content (μg/g)	*K*_m_ (mmol/L)
Caffeic acid	81.92 ± 5.34b	6.67 ± 0.74e
Epigallocatechin	28.63 ± 2.11c	9.14 ± 1.33 cd
Catechin	112.89 ± 7.94a	7.84 ± 0.89de
Epicatechin	14.71 ± 1.36e	10.45 ± c
Dimethyl ellagic acid	16.53 ± 2.78e	12.86 ± 1.34a
Esculin	21.38 ± 2.13d	10.67 ± 1.21ab

*Note:* Means with different letters in the same column are significantly different using one‐way ANOVA followed by the Duncan's test, *p* < 0.05.

The affinity of PPO for these phenolic substrates was evaluated by the Michaelis constant (*K*_m_), where a lower *K*_m_ indicated stronger affinity (Table 2). Caffeic acid and catechin exhibited the lowest *K*_m_ values (6.67 ± 0.74 mmol/L and 7.84 ± 0.89 mmol/L, respectively), suggesting the highest affinity. In contrast, dimethyl ellagic acid showed the highest *K*_m_ (12.86 ± 1.34 mmol/L). Combining the content of phenolic substrates, the binding energy, and the affinity *K*_m_ value of PPO, it was speculated that catechin and caffeic acid were the specific phenolic substrates of PPO in the lotus root. Differences in variety and cultivation origin led to variations in the predominant phenolic substrates in lotus root, which in turn significantly affected their binding affinity with PPO. Furthermore, caffeic acid and catechin have also been identified as optimal substrates for PPO in other species such as dendrobium officinale flowers and apricot (Derardja et al. 2022; Zhong et al. 2025).

### 3.5 Binding Sites Analysis Between PPO and Its Specific Substrates

The results of molecular docking between PPO and the specific phenolic substrates are presented in Figure 3B,C. The interaction diagram for caffeic acid (Figure 3B) reveals its binding mode with PPO and shows the formation of 5 hydrogen bonds with residues ASP54, ALA52, ILE46, ALA137, and THR135 of PPO and formed hydrophobic interactions with ILE46 and PRO48. Notably, ILE46 established both hydrogen bonds and hydrophobic contacts with caffeic acid, suggesting that this residue plays a pivotal role in stabilizing substrate binding and may contribute to the substrate specificity of lotus root PPO.

**FIGURE 3:**
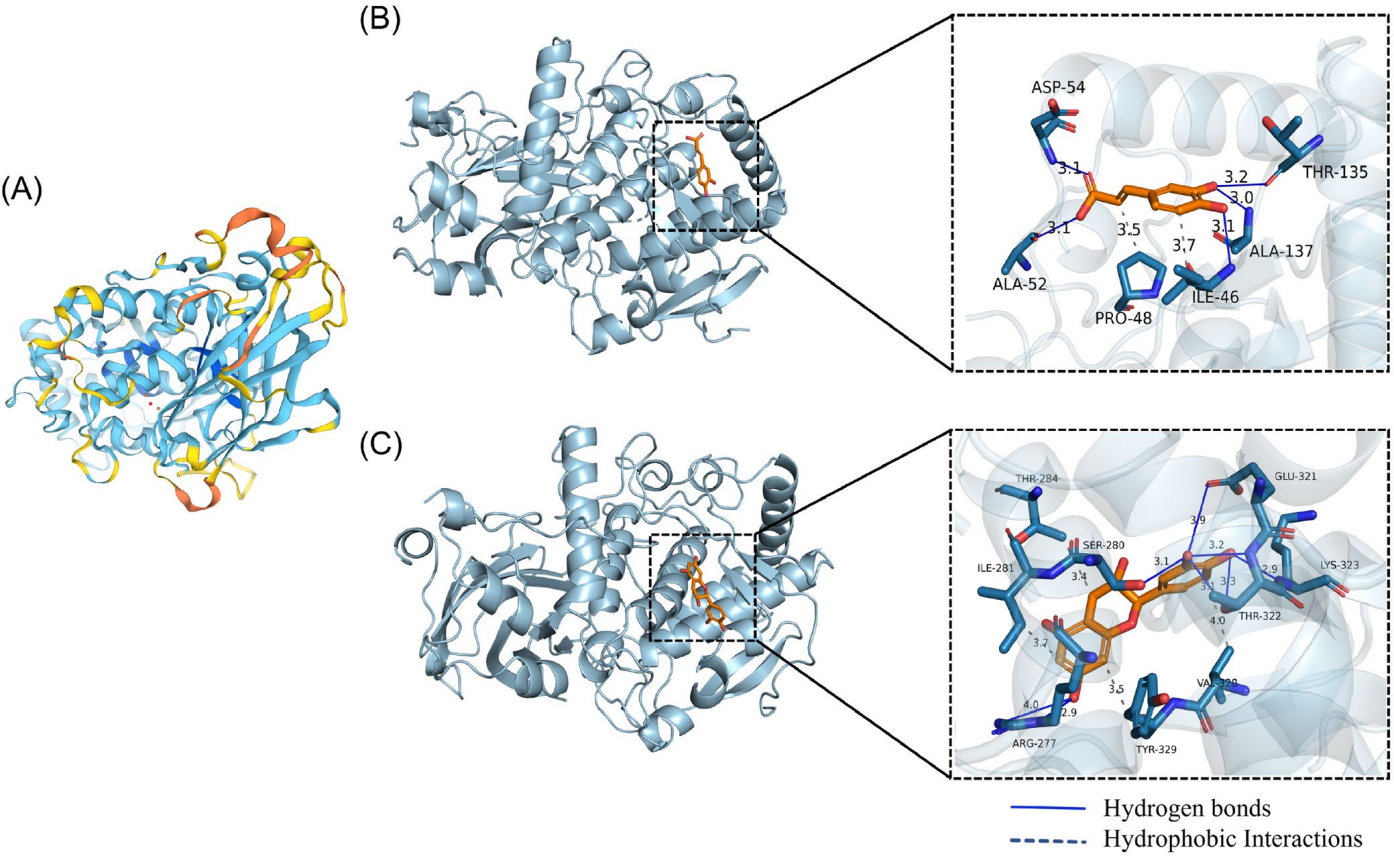
The molecular docking between lotus root PPO and the specific phenolic substrates. (A) The 3D structure of lotus root PPO; (B) the interaction diagram of molecular docking between PPO and caffeic acid; (C) the interaction diagram of molecular docking between PPO and catechin.

The molecular docking result of PPO with catechin is shown in Figure 3C. The results reveal that 8 hydrogen bonds are formed between catechin and the amino acid residues SER280, ARG277, THR322, LYS323, and GLU321 of PPO. In detail, ARG277 formed 2 hydrogen bonds, and THR322 forms 3 hydrogen bonds with the hydroxyl groups of (+)‐catechin, respectively. The hydrogen bonds might be a direct and critical force inducing conformational changes in PPO, thereby enhancing the catalytic activity of PPO on catechin. This molecular mechanism provided an explanation for the strong binding affinity and the low docking energy observed between PPO and catechin. The docking mode of PPO from *Nelumbo* genus varied even for stereoisomeric substrates, and hydrogen bonds played a key role in the binding mode of PPO (Dong et al. 2017; Li et al. 2021).

### 3.6 Inhibitory Effects of Inhibitors on Fresh‐Cut Lotus Root Slices

Based on previous investigations into the catalytic mechanism of PPO from lotus root (Li et al. 2023, 2021), PPO exhibited the strongest affinity for flavanols such as catechin, epicatechin, and epigallocatechin. This suggested the preference of *Nelumbo* PPO for flavanol compounds. Accordingly, flavanol derivatives structurally similar to these specific substrates were selected as potential competitive inhibitors. As WT is rich in these flavanol derivatives, the WT extract was selected for PPO inhibition studies. In addition, Vc, a common browning inhibitor, was used as a positive control. As shown in Figure 4A, both inhibitors, WT extract and Vc, effectively reduced the activity of PPO in a concentration‐dependent manner (*p* < 0.05). At a concentration of 5 mM, Vc inhibited PPO activity to 46.79% ± 5.67% of the control, while WT extract resulted in a relative activity of 39.86% ± 3.34%. When the concentration was increased to 10 mM, the inhibitory effect was more pronounced, with relative activities decreased to 24.33.86% ± 1.06% for WT extract and 28.58% ± 3.94% for Vc. These data revealed that WT extract was a potent PPO inhibitor, as it consistently led to lower residual activity at both concentrations.

**FIGURE 4:**
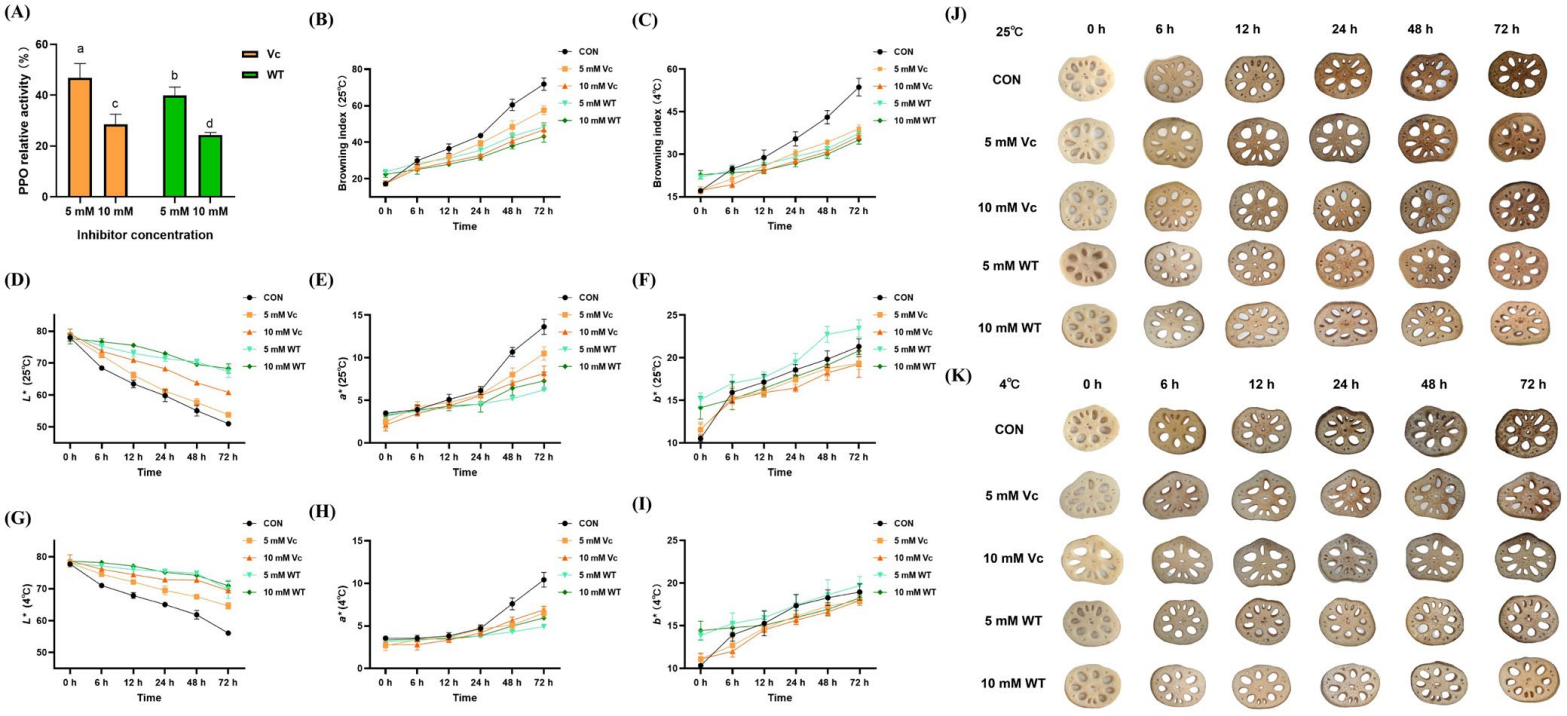
Inhibition effects of WT extract and Vc on the browning of lotus root. (A) PPO relative activity; (B) Browning index of lotus root slices at 25°C; (C) Browning index of lotus root slices at 4°C; (D) The L* values of lotus root slices at 25°C; (E) The a* values of lotus root slices at 25°C; (F) The b* values of lotus root slices at 25°C; (G) The L* values of lotus root slices at 4°C; (H) The a* values of lotus root slices at 4°C; (I) The b* values of lotus root slices at 4°C; (J) Fresh‐cut lotus root slices treated with inhibitors during storage at 25°C. (K) Fresh‐cut lotus root slices treated with inhibitors during storage at 4°C. Bars with various lowercase letters differ significantly using one‐way ANOVA followed by the Duncan's test, *p* < 0.05.

The effects of WT extract and Vc on inhibiting browning in fresh‐cut lotus root slices are presented in Figure 4B–K. Under 25°C conditions, the WT extract treatment group significantly inhibited the browning index of lotus root, particularly the 10 mM WT treatment, which notably suppressed the degree of browning (Figure 4B). The browning index increased most slowly in this group, demonstrating a better effect than the Vc treatment group. Specifically, the WT treatment group effectively delayed the decrease in surface brightness (L* value) and the increase in a* value of lotus root slices (Figure 4D,E). Moreover, higher concentrations of WT showed more pronounced inhibitory effects on the browning index of lotus root. In contrast, Vc, as one of the most common browning inhibitors, was less effective than the WT treatment group in suppressing lotus root browning but performed better in inhibiting the rise in b* value (Figure 4D–F).

Similarly, during storage at 4°C, WT treatment effectively delayed the decrease in L* value and the increase in a* value of lotus root slices, with its inhibitory effect outperforming that of the Vc treatment group (Figure 4C,G,H). This indicated that WT treatment exhibited excellent efficacy in maintaining the brightness of fresh‐cut lotus root slices and suppressing their tendency to turn red or brown. The Vc demonstrated a better effect in inhibiting the increase in b* value of lotus root slices (Figure 4I). In addition, images of lotus root slices treated with inhibitors and stored at 25°C and 4°C are presented in Figure 4J,K, respectively. These differences highlighted the distinct performances of the traditional color‐preserving agent the WT extract and Vc in inhibiting lotus root browning. The main component of WT extract, flavanol alkaloids, contains multiple hydroxyl groups and has strong free radical scavenging and reducing abilities, which can effectively inhibit lotus root browning. Similarly, a previous study found that purslane extract significantly suppressed the activities of PPO, which was inseparable from the polyphenols and alkaloids present in purslane (Liu et al. 2019).

### 3.7 The Impact of Lotus Root Treated With Inhibitors on Gut Microbiota

As shown in Figure 5, the effects of lotus root slices treated with different inhibitors on gut microbiota were assessed using an in vitro fecal fermentation model. The microbial community in the WT treated lotus root group and Vc treated lotus root group differed significantly from those in the untreated control (CON) group (Figure 5A–C). Notably, compared to the CON group, WT treatment markedly enhanced the α diversity of the gut microbiota, as indicated by increases in the Chao 1, Shannon, and ACE index. These findings suggest that WT treated lotus root significantly enriched the diversity of gut microbiota.

**FIGURE 5:**
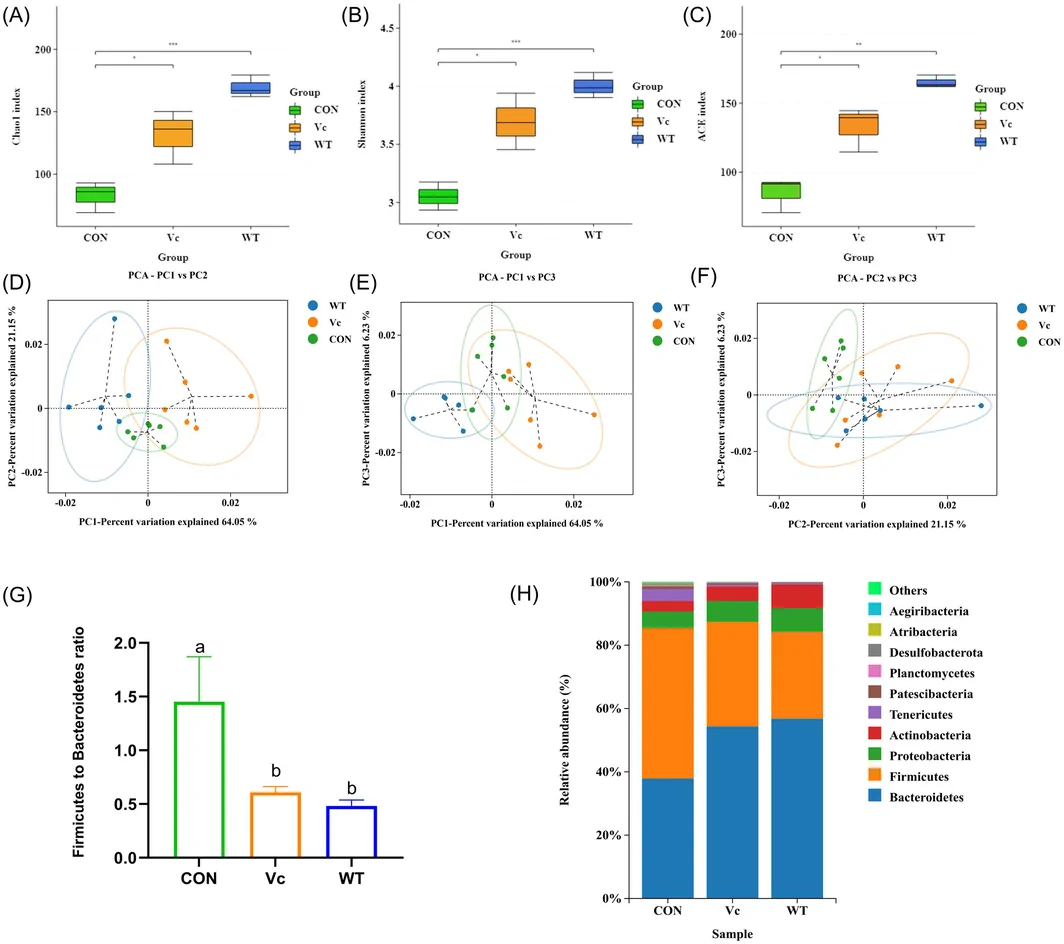
The effects of lotus root slices treated with different inhibitors on gut microbiota. (A) Chao1 index of α‐diversity; (B) Shannon index of α‐diversity; (C) ACE index of α‐diversity; (D–F) PCA analysis; (G) *Firmicutes*/*Bacteroidetes* ratio; (H) Relative abundance of dominant gut microbiota species at the phylum level. Bars with various lowercase letters differ significantly using one‐way ANOVA followed by the Duncan's test, *p* < 0.05.

Beta diversity analysis revealed that both WT treatment and Vc treatment induced notable shifts in the gut microbiota, as demonstrated by PCA (Figure 5D–F). The results indicated that lotus root treated with these inhibitors altered the impact of lotus root consumption on gut microbial composition. Specifically, both WT extract and Vc treatments exhibited significant effects on reducing the ratios of *Firmicutes*/*Bacteroidetes* (*F*/*B*) (Figure 5G, *p* < 0.05). At the phylum level, *Bacteroides* and *Firmicutes* were found to be the predominant phyla among 10 phyla across all groups (Figure 5H). The *F*/*B* ratio is a crucial indicator for assessing the structure of the gut microbial community, and the changes in *F*/*B* ratio are closely associated with various health conditions including gut dysbiosis, obesity, diabetes, etc. A decrease in the *F/B* ratio typically implies a potentially healthier gut microbiota profile, characterized by improved metabolic health, and a lower risk associated with conditions like obesity and inflammatory bowel disease (Stojanov et al. 2020; Vishwakarma et al. 2026).

As shown in Figure 6A, linear discriminant analysis effect size (LEfSe) was used to analyze differentially abundant bacterial taxa across groups. Distinct microbial biomarkers were observed for the CON, WT, and Vc groups (Figure 6B). Specifically, 26 biomarkers were unique to the CON group, 8 to the Vc group, and 11 to the WT group. The *p_Firmicutes* represented the most prominent biomarker in the CON group. The WT group demonstrated a marked enrichment in the relative abundance of *g_Bifidobacterium*, a genus recognized for its antioxidant and anti‐inflammatory properties (Dong et al. 2022; Sun et al. 2024), as well as its ability to improve glycemic control, reduce blood lipid levels, and enhance immune function. For the Vc group, *f_Bacteroidaceae* exhibited the highest linear discriminant analysis (LDA) score. These findings suggest that consumption of lotus root treated with WT effectively modulated gut microbiota composition, suggesting potential health‐promoting benefits.

**FIGURE 6:**
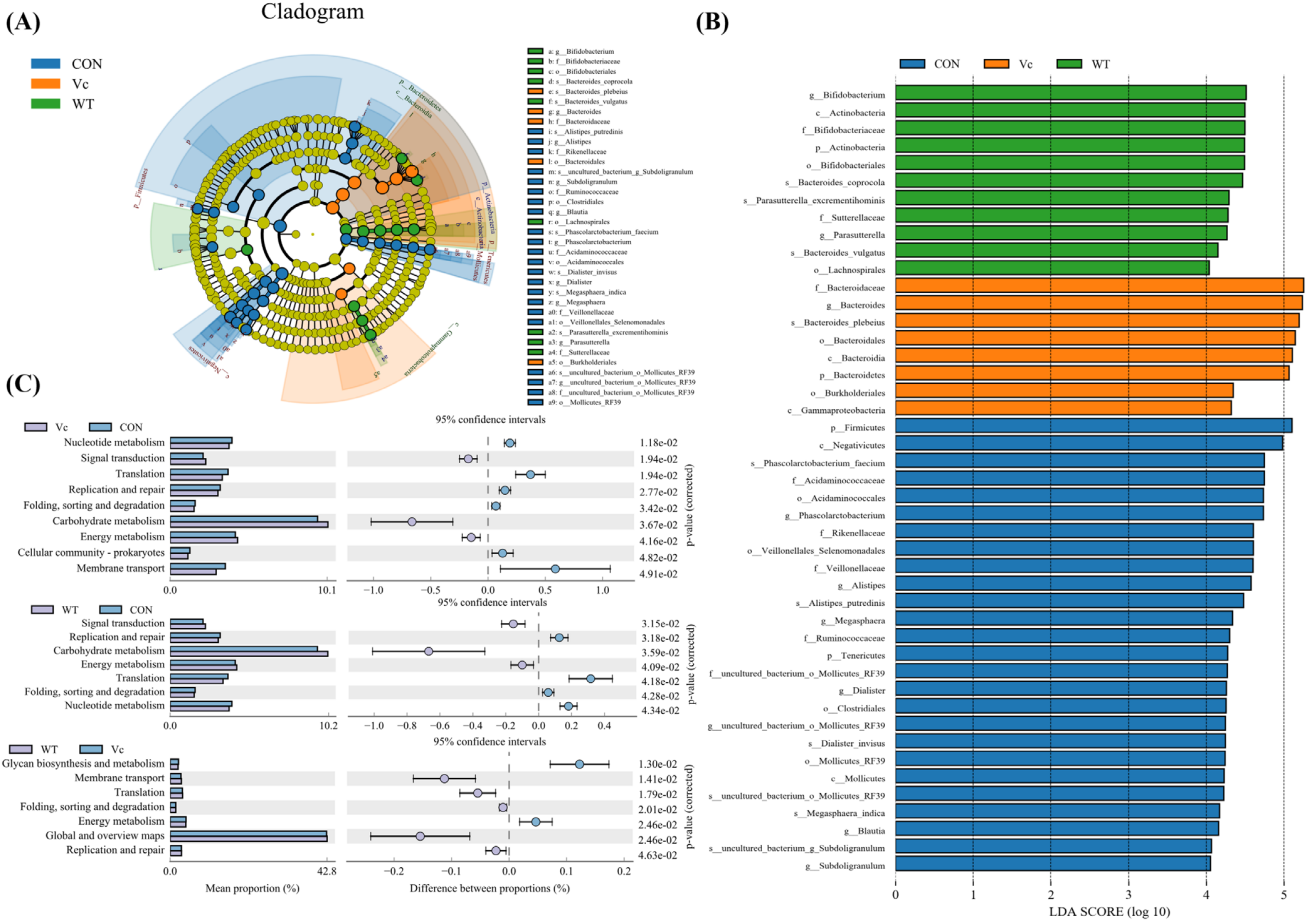
Effects of lotus root slices treated with different inhibitors on LEfSe analysis and KEGG pathway enrichment analysis. (A) LEfSe analysis; (B) Distribution histogram based on LDA; a higher LDA score represents greater importance of the bacteria; (C) KEGG pathway enrichment analysis.

KEGG metabolic pathway analysis was performed to compare metabolic functional profiles across the CON group, Vc group, and WT group. Compared to the CON group, the Vc group exhibited significant alterations in 9 pathways, primarily involving membrane transport, carbohydrate metabolism, translation, and signal transduction (Figure 6C, *p* < 0.05). The WT group showed significant differences in 7 pathways compared to the CON group, including carbohydrate metabolism, translation, signal transduction, replication and repair, and nucleotide metabolism. Furthermore, 7 pathways differed significantly between the WT and Vc groups, mainly related to glycan biosynthesis and metabolism, membrane transport, and energy metabolism. Vc, as an antioxidant, broadly affecting oxidative stress‐sensitive microorganisms and enriching those adapted to such conditions, resulted in the modulation of pathways including membrane transport and signal transduction. For WT extract, which primarily consists of flavanol alkaloids, it operated through a more complex mechanism due to its combined alkaloid and polyphenol properties. The phenolic hydroxyl structures of flavanol alkaloids showed an antioxidant mode distinct from that of Vc and enable direct interactions with microbial membranes or proteins. Consequently, WT influenced specific pathways such as energy metabolism and glycan biosynthesis. These results showed that the WT and Vc differentially shape the functional profile of the gut microbiota and produced different metabolic characteristics.

The mechanistic link between PPO inhibition and gut microbiota modulation was mediated through the preservation of phenolic compounds and the modulatory effect of WT on the gut microbiota (Sui et al. 2023). In fresh‐cut lotus root, PPO catalyzed the oxidation of phenolic substrates to reactive quinones, which caused both browning and a substantial reduction in phenolic content. By inhibiting PPO activity, the structural integrity of polyphenols in lotus root was preserved, allowing them to reach the colon where the gut microbiota metabolized polyphenols into bioactive metabolites (Li, Luo, et al. 2025; Li, Nie, et al. 2025; Li, Zheng, et al. 2025). These metabolites exerted a prebiotic‐like effect by promoting beneficial bacteria including *Bifidobacterium* and *Lactobacillus*, while inhibiting pathogenic species (Gowd et al. 2019). Moreover, preventing oxidation helped maintain the carbohydrate structure of dietary fiber and resistant starch in lotus root, both of which serve as fermentable substrates for beneficial microorganisms in the colon. Browning inhibition preserved phenolic compounds that can form inclusion complexes with starch, thereby enhancing starch's resistance to enzymatic digestion through both direct inhibition of α‐amylase and structural reorganization of starch molecules (Fan et al. 2025). Regarding the reduction of harmful or opportunistic bacteria, flavonol derivatives in WT had been reported to neutralize ROS, regulate gut microbiota imbalances, improve the relative abundance of *Bacteroidetes*, and reduce the relative abundance of *Proteobacteria* (Zhou et al. 2025). In addition, phenolic compounds from lotus root also demonstrated antimicrobial activity against pathogenic strains. Gut microbiota metabolized bioactive compounds from WT and lotus root into metabolites with enhanced antioxidant activity, which may further support intestinal homeostasis (Annunziata et al. 2018). Such metabolites may further promote the growth of beneficial bacteria, while suppressing potentially pathogenic taxa, thereby contributing to the prebiotic effects of WT‐treated lotus root. Therefore, these findings indicated that PPO inhibition not only delayed browning but also preserved the bioactive phenolic profile of lotus root, thereby contributing to gut microbiota modulation.

## 4 Discussion

The enzymatic browning of fresh‐cut lotus root, primarily mediated by PPO, presents a significant challenge to its shelf‐life and sensory quality. These are two key issues in the study of lotus root browning. On the one hand, exploring the mechanism by which PPO catalyzes phenolic substrates leading to browning in lotus roots is of great significance. On the other hand, it is necessary to find suitable natural products as inhibitors to delay the browning of fresh‐cut lotus root and explore the effects of lotus root treated with inhibitors on regulating gut microbiota.

In the present study, the browning pattern of PPO in the fresh‐cut slices of “Cizhou” white lotus root were explored, and the browning pattern of lotus roots varies depending on the variety and place of origin. The changes indicated that the lotus root slices were mainly decreased in brightness and increased in yellow value at the early stage of browning, whereas a significant rise in redness was observed after 24 h. Thus, it is speculated that PPO preferentially catalyzed certain substrates at the early phase, producing browning products that are predominantly showed the color of yellow. In the later stage, the catalytic substrates changed, leading to a marked increase in the red component. Previous studies confirmed that PPO preferentially recognized and catalyzed specific substrates (Li et al. 2023). The browning pattern of lotus root was highly correlated with its internal active PPO and abundant endogenous phenolic substrates. The PPO from lotus root was purified and identified, and the 3D structure of PPO was established, which helped understanding the catalytic properties of lotus root PPO. In addition, the composition and content of phenolic compounds were analyzed, and the results revealed that catechin and caffeic acid are the most abundant phenolic compounds in lotus root. It was particularly noteworthy that these two main endogenous phenolic substrates had the highest affinity with PPO of lotus root, which may be the key reason why lotus root was prone to browning. Furthermore, docking studies indicated that the strong binding affinity of PPO toward catechin and caffeic acid was primarily attributed to the formation of multiple hydrogen bonds. Nevertheless, catechin and caffeic acid exhibited differences in the number of hydrogen bonds and their binding sites with PPO, which may be due to changes in their molecular structure, spatial conformation, and number of hydroxyl groups. caffeic acid had the smaller molecular size, lower steric hindrance, and the presence of a carboxyl group in caffeic acid. Although for catechin, a flavan‐3‐ol, its relatively compact structure facilitated entry into the PPO active cavity and allowed effective binding to the catalytic residues, thus PPO exhibited strongest affinity for catechin. Catechin formed robust hydrogen bonds with key residues within the active sites of PPO, which significantly contributed to the stability of the enzyme‐substrate complex and explained the high binding affinity observed (Cai et al. 2023). In addition, PPO from different sources differs in its substrate specificity. For example, PPO from recombinant pea seed coat‐specific displayed the highest affinity for catechols (Franková et al. 2025); The key substrates of pineapple PPO were identified as catechin, epicatechin, chlorogenic acid and pyrocatechol (Liu, Li, et al. 2024); PPO from granny smith apple exhibited the substrate preference for catechol and 4‐methylcatechol (Han et al. 2019). Chlorogenic acid was the main endogenous substrate of goji berries enzymatic browning (Gao et al. 2024). The in‐depth analysis of the binding sites and binding forces of lotus root PPO clarified that the role of key molecular forces hydrogen bonds in substrate recognition and binding, and explained the catalytic preference of PPO from the molecular level and in‐depth mechanistic. However, not all phenolic compounds serve as equivalent substrates for PPO, and subtle structural differences significantly influence their binding modes and catalytic efficiency. The present study demonstrated that PPO substrate selectivity was primarily governed by molecular size, the number and position of hydroxyl groups, and the spatial conformation of phenolic compounds. The phenolics with low molecular weight such as catechin and caffeic acid were identified as more efficient PPO substrates, which is attributable to their better geometric compatibility with the PPO active sites. Previous study employed Gaussian‐accelerated molecular dynamics simulations to investigate the catalytic efficiency mechanisms of lotus root PPO with specific substrates including catechin, and revealed that upon substrate binding, the α‐helix in the Q53–D63 region near the copper ion underwent extension, which stabilized the active sites and enhanced catalytic efficiency (Liu, Zheng, et al. 2024). In tomato PPO, the gatekeeper residue Phe270 located at the entrance of the active site has been shown to selectively stabilize or hinder the entry of specific substrates through distinct side‐chain orientations (Kampatsikas et al. 2019). These substrate‐induced conformational changes directly modulated catalytic efficiency. Thus, the inherent phenolic profile, particularly the presence and relative abundance of specific substrates such as catechins and caffeic acid was a critical determinant of browning susceptibility, and the rate and extent of browning were governed by the interplay between substrate composition, PPO conformational dynamics, and environmental factors such as temperature.

Moreover, the browning of fresh‐cut lotus root is associated with multiple factors, encompassing transcriptional regulation of PPO, synergistic interactions with other oxidative enzymes such as peroxidase, temperature dependence, and other environmental and physiological parameters. Dong et al. (2016) isolated the complete cDNA of NnPPO from lotus and confirmed that transcriptional activation of NnPPO was probably the main reason causing lotus root browning. Beyond PPO, POD and ROS function as synergistic enhancers in the browning process. The activities of PPO and POD, along with the expression of their encoding genes (NnPPOA and NnPOD), increase concurrently with browning progression. The upregulation of NnPPOA and NnPOD by high temperature coincided with the increase in related enzyme activities and the browning degree, identifying these as the most important candidate genes for fresh‐cut lotus root browning (Min et al. 2017). ROS, generated as byproducts of wounding and mitochondrial respiration, can oxidize phenolic compounds or promote browning by enhancing PPO activity (Shi, Xie, Li, et al. 2024). The interplay among these three factors constituted an oxidative cascade that conferred predictable kinetic characteristics to the browning process.

At present, the widely used PPO browning inhibitors still have great limitations, such as H_2_S, organic acids, and other commonly used inhibitors that are sensitive to light and heat, and there are still many concerns about their side effects, seriously restricting the application in the processing and storage of lotus roots. Consequently, the search for suitable inhibitors from natural extracts is of particular importance. In this present study, WT extract was selected due to its reported high content of flavanol derivatives, especially flavanol alkaloids, which are structurally similar to the key substrate catechins in lotus root. This study confirmed the promising efficacy of WT extract in inhibiting PPO activity. Compared to the normal inhibitor Vc, WT more effectively suppressed browning in fresh‐cut lotus root and demonstrated a significant capacity to maintain slice brightness and mitigated increases in red value. These results suggested that the main mechanism of WT extract involved the antioxidant capacity of its key active ingredients and effective inhibition of PPO activity, thereby reducing the generation and accumulation of quinones and delaying the core browning reaction responsible for the loss of brightness and emergence of reddish‐brown discoloration. A previous study confirmed that WT extract showed antioxidative efficacy, and there was potential utility as a preventive measure to limit oxidative damage (Rangi et al. 2018). Plant‐derived flavonoid alkaloids have been shown to inhibit enzymatic browning caused by PPO. For instance, citrus alkaloids suppressed PPO activity in lychee pericarp, expanding their potential application in the postharvest preservation of fruits and vegetables. Furthermore, various bioactivities of flavonoid alkaloids have been reported. Wang et al. (2014) found that flavanol derivatives isolated from Pu‐erh tea protected cells from H_2_O_2_‐induced damage. The esterified flavonoid alkaloids from WT significantly inhibit the formation of advanced glycation end products (Li et al. 2018). In addition, cinnamoyl flavonoid alkaloids from green tea have demonstrated strong inhibitory activity against acetylcholinesterase and exhibited anti‐aging effects (Ke et al. 2024).

Most studies have only explored the effect of inhibitors on browning of lotus root, however, the potential impact of consuming inhibitor‐treated lotus root on the gut microbiota remains unexplored. The present study showed that the lotus root treated with the natural extract WT, which was rich in flavanol derivatives mainly including 4 flavoalkaloids: etc‐pyrrolidinone A, etc‐pyrrolidinone B, etc‐pyrrolidinone C, and etc‐ pyrrolidinone D, had a positive effect on the gut microbiota. Notably, the flavanol alkaloids present in the WT extract have been reported to reduce reactive oxygen species accumulation and improve intestinal health by enhancing gut microbial homeostasis, increasing the production of short‐chain fatty acids (SCFAs), and reducing fat deposition (Ke et al. 2024). These properties suggested a role for WT treatment in both preserving postharvest quality of fresh lotus root slices and potentially improving host physiology through microbiota‐mediated mechanisms. The results of present study further revealed that ingestion of WT‐treated lotus root significantly modulated the gut microbiota, as evidenced by improved microbial diversity, an increased *F*/*B* ratio, and a marked enrichment in the relative abundance of the probiotic *g_Bifidobacterium*. The human gut microbiota is overwhelmingly dominated by two major phyla, *Firmicutes* and *Bacteroidetes*, together accounting for over 90% of the total bacterial community (Eckburg et al. 2005). In particular, *Bifidobacterium* exerts potent pro‐homeostatic and anti‐inflammatory effects, as well as strengthening intestinal barrier function (Gavzy et al. 2023). Meanwhile, lotus root polyphenols mainly consist of catechins and flavoalkaloids are characteristic compounds that accumulate in WT, so both of WT and lotus root are rich in phenolic hydroxyl groups, providing a common molecular basis for antioxidant activity through free radical scavenging, metal chelation, and activation of endogenous antioxidant defense systems. Moreover, WT‐derived flavoalkaloids and lotus root polyphenols exert synergistic prebiotic effects by neutralizing ROS, inhibiting harmful bacteria e.g., *Bacteroides*, *Escherichia‐Shigella*, promoting beneficial genera including *Akkermansia*, *Lactobacillus*, and *Bifidobacterium*, restoring microbial balance, and elevating SCFA levels (Lin et al. 2024). Therefore, the enrichment of *Bifidobacterium* and suppression of opportunistic pathogens observed in WT‐treated group are consistent with the known effects of WT and its characteristic flavoalkaloids in inhibiting harmful bacteria. These findings indicated that WT treatment not only served as an effective inhibitor to delay browning in fresh‐cut lotus root but also offered potential prebiotic‐like benefits by modulating gut microbiota composition.

In this study, PPO inhibition preserved phenolic compounds of lotus root from enzymatic oxidation during storage. These phenolics are metabolized by the gut microbiota, which encoded a diverse array of phenolic‐associated enzymes including tannase, phenolic acid reductase, etc. (Rodríguez‐Daza et al. 2021). It has been previously reported that phenolic‐transforming bacteria, such as *Bifidobacterium* and *Lactobacillus*, thrive when provided with phenolic substrates, and convert phenolic compounds into bioactive metabolites (Rakhra et al. 2026). These metabolites modulate gut pH, inhibit pathogen adhesion, act as signaling molecules that regulate host immune responses and intestinal barrier function, and this process contributes to the maintenance of gut homeostasis. Thus, PPO inhibition by WT preserved phenolic substrates for gut microbial metabolism, linking anti‐browning treatments to prebiotic functionality.

In addition, preventing oxidation is critical for preserving carbohydrate integrity of lotus root. Oxidation disrupts the ordered structure of dietary fiber and resistant starch in fresh‐cut lotus root via reactive quinones and ROS, weakening their enzymatic resistance. Notably, starch and polyphenols can form inclusion complexes through non‐covalent interactions, which enhance enzyme resistance and enable gradual polyphenol release to inhibit α‐amylase (Deng et al. 2025). In vitro fermentation confirmed that starch‐polyphenol complexes promoted beneficial bacteria such as *Bifidobacterium* and *Lactobacillus* while suppressing pathogens (Zhu et al. 2025). Preventing oxidation in fresh‐cut lotus root processing not only protected the bioactivity of phenolic compounds but also facilitated the formation of starch‐polyphenol complexes, which may further enhance prebiotic effects.

The study investigated the anti‐browning, PPO inhibitory, and prebiotic potentials of WT extract on lotus root slices, while several limitations should be acknowledged. The prebiotic potential was assessed using an in vitro fecal fermentation model while in vivo studies are warranted to confirm the physiological relevance of the observed gut microbiota modulation. Future research should focus on the prebiotic activity of WT extract on lotus root slices in vivo as well as evaluating the long‐term stability and sensory quality of WT‐treated lotus root products. Such efforts would support the development of novel lotus root‐based products that combine anti‐browning properties with enhanced probiotic functions, thus extending shelf life and delivering additional health benefits to consumers.

## 5 Conclusion

In conclusion, this study systematically elucidated the enzymatic browning mechanism of fresh‐cut lotus root and demonstrated the dual functionality of WT extract as both an anti‐browning agent and a prebiotic modulator. The browning process followed specific kinetics, with L* and b* values changing most rapidly within the first 6 h and a* value increasing markedly after 24 h. Lotus root PPO (sequence D6C6L2) was identified and its 3D structure established, and molecular docking studies revealed that the strong binding affinities of PPO toward specific substrates, catechin and caffeic acid, were primarily attributed to the formation of 8 and 5 hydrogen bonds, respectively. Moreover, WT extract rich in flavanol derivatives effectively reduced the activity of PPO in a concentration‐dependent manner and inhibited browning in fresh‐cut lotus root slices. In an in vitro fermentation model, WT‐treated lotus root enhanced gut microbiota diversity, reduced the *F*/*B* ratio, enriched *Bifidobacterium*, and modulated carbohydrate and glycan metabolism pathways, indicating significant prebiotic potential. This study provided the foundation for the development of lotus root products with integrated fresh‐keeping and probiotic functions, bridging the gap between postharvest preservation and functional food development. Future research should focus on in vivo validation of the prebiotic efficacy and identification of the specific bioactive compounds responsible for the observed synergistic effects.

## Author Contributions

**Jingfang Li:** conceptualization, data curation, formal analysis, writing – original draft, project administration, funding acquisition, writing – review and editing, validation, resources. **Chuangkai Xing:** investigation, methodology, software, data curation, writing – review and editing, visualization. **Fan Jiang:** methodology, data curation. **Xiaoru Liu:** supervision, writing – review and editing.

## Funding

This work was supported by the Natural Science Foundation of Hebei Province, C2024402020. Science Research Project of Hebei Education Department in Hebei Province, China, BJ2025099. Hebei University of Engineering “QingLan” Scholars Launch Fund and Innovation Fund, China.

## Conflicts of Interest

The authors declare no conflicts of interest.

## Supporting information


**Figure S1:** The reliability of PPO structure verified via Ramachandran plots.
**Table S1:** Identification of the PPO purified from lotus root.
**Table S2:** Binding energies of molecular docking for PPO and major phenolic compounds in lotus root.

## Data Availability

The data that support the findings of this study are available from the corresponding author upon reasonable request.
