# Supplementary material for: Anti‐Browning and Prebiotic Effects of White Tea Extract on Fresh‐Cut Lotus Root
Source: Food Sci Nutr. 2026 Oct 9;14(10):e72464. doi: 10.1002/fsn3.72464 (PMC13653074; doi:10.1002/fsn3.72464)
Supplement: Supplementary file 1 [file FSN3-14-e72464-s001.docx]

**Supplementary material**

**Figure S1.** The reliability of PPO structure verified via ramachandran plots

**Table S1.** Identification of the PPO purified from lotus root.

| Accession | Description | PepCount | Unique PepCount | Cover Percent | MW  [kDa] | PI |
| --- | --- | --- | --- | --- | --- | --- |
| D6C6L2 | tr\|D6C6L2\|D6C6L2_NELNU Polyphenol oxidase OS=Nelumbo nucifera OX=4432 GN=PPOA PE=2 SV=1 | 2 | 2 | 2.35% | 67.22 | 8.59 |

**Table S2**. Binding energies of molecular docking for PPO and major phenolic compounds in lotus root.

| No. | Ligands | Molecular formula | Binding energy  (kcal/mol) |
| --- | --- | --- | --- |
| 1 | Caffeic acid | C_9_H_8_O_4_ | -5.73 |
| 2 | 5-*p*-Coumaroylquinic acid | C_12_H_18_O_11_ | 3.65 |
| 3 | Rutin | C_23_H_30_O_19_ | 479.90 |
| 4 | Hyroxyproanthocyanidin dimers B1-OH | C_30_H_26_O_13_ | 378.79 |
| 5 | Hyroxyproanthocyanidin dimers B2-OH | C_30_H_26_O_13_ | 389.44 |
| 6 | Proanthocyanidin B3 | C_30_H_26_O_12_ | 201.57 |
| 7 | Hyroxyproanthocyanidin dimers B4-OH | C_30_H_26_O_13_ | 871.87 |
| 8 | Epigallocatechin | C_15_H_14_O_7_ | -2.48 |
| 9 | Catechin | C_15_H_14_O_6_ | -6.99 |
| 10 | Epicatechin | C_15_H_14_O_6_ | -2.63 |
| 11 | Dimethyl ellagic acid | C_14_H_18_O_9_ | -1.49 |
| 12 | Swertianolin | C_21_H_24_O_10_ | 7.62 |
| 13 | Esculin | C_23_H_32_O_2_ | -2.09 |
